# A healthy turn in urban climate change policies; European city workshop proposes health indicators as policy integrators

**DOI:** 10.1186/1476-069X-11-S1-S14

**Published:** 2012-06-28

**Authors:** Hans Keune, David Ludlow, Peter van den Hazel, Scott Randall, Alena Bartonova

**Affiliations:** 1 Research Institute for Nature and Forest (INBO), Brussels; Centre of Expertise for Environment and Health, Faculty of Political and Social Sciences, University of Antwerp; naXys, Namur Center for Complex Systems, University of Namur, Belgium; 2 Centre for Sustainable Planning and Environments, University of the West of England, Bristol, U.K. - formerly Bristol City Planning Department; 3 Public Health Services Gelderland-Midden, Arnhem, The Netherlands; 4 NILU – Norwegian Institute for Air Research, Kjeller, Norway

## Abstract

**Background:**

The EU FP6 HENVINET project reviewed the potential relevance of a focus on climate change related health effects for climate change policies at the city region level. This was undertaken by means of a workshop with both scientists, city representatives from several EU-countries, representatives of EU city networks and EU-experts. In this paper we introduce some important health related climate change issues, and discuss the current city policies of the participating cities.

**Methods:**

The workshop used a backcasting format to analyse the future relevance of a health perspective, and the main benefits and challenges this would bring to urban policy making.

**Results:**

It was concluded that health issues have an important function as indicators of success for urban climate change policies, given the extent to which climate change policies contribute to public health and as such to quality of life. Simultaneously the health perspective may function as a policy integrator in that it can combine several related policy objectives, such as environmental policies, health policies, urban planning and economic development policies, in one framework for action. Furthermore, the participants to the workshop considered public health to be of strategic importance in organizing public support for climate change policies. One important conclusion of the workshop was the view that the connection of science and policy at the city level is inadequate, and that the integration of scientific knowledge on climate change related health effects and local policy practice is in need of more attention. In conclusion, the workshop was viewed as a constructive advance in the process of integration which hopefully will lead to ongoing cooperation.

**Conclusions:**

The workshop had the ambition to bring together a diversity of actor perspectives for exchange of knowledge and experiences, and joint understanding as a basis for future cooperation. Next to the complementarities in experience and knowledge, the mutual critical reflection was a bonus, as ideas had the opportunity to be scrutinized by others, leading to more robustness and common ground. The structured backcasting approach was helpful in integrating all of this with one common focus, embracing diversity and complexity, and stimulating reflection and new ideas.

## Background

### Introduction

In this paper we report how several developments and insights relevant to climate change came together in one workshop. 1) The growing concern for climate change related health effects and simultaneously the idea that public health effects from a strategic point of view may be of growing importance for awareness raising about the need for climate change policies. 2) The growing awareness that next to natural scientific knowledge about climate change (and health effects) also social scientific knowledge on climate change related processes is of key importance for tackling the challenges ahead. 3) The notion that transdisciplinarity (scientific - non-scientific actor cooperation) is important in sustainability problem solving processes. 4) The foresight that worldwide more than half of the global population in future will be living in urban areas. In an event organized by an interdisciplinary HENVINET team these insights came together in a backcasting workshop in which urban management experts and environment and health scientists discussed the need for combining the city management and health perspectives in relation to climate change policies.

### 1) Climate change and the strategic nature of public health

Vineis [1] discusses what he calls *“the newborn science of health effects of climate change”*. Newborn as the scientific challenges of climate change related health effects surpass the capacities of current main stream epidemiology. The complex entanglement of climate change with other elements influencing public health, makes it very difficult to distinguish climate change related health effects from other causes. Even mental health effects may directly or indirectly be attributed to climate change, e.g. as a consequence of wars and conflicts caused by climate change related developments. The methodological challenges are huge [2], but still quite some health impacts are currently attributed directly or indirectly to climate change [2,3]. A main effect from climate change which will probably have big impact on human health is the rising temperature. *“Since its first brief assessment of climate change and health in 1989 the World Health Organization* (*WHO*) *has published several substantial reviews highlighting growing concern at the international level*, *including a recent call for intensified research efforts* (*WHO 2009*)*. In 2008*, *a World Health Assembly* (*WHA*) *resolution on Climate Change and Health was adopted* (*WHA61.19*)*. The consensus is that large populations will be affected by extreme weather situations*, *heat stress*, *water and food scarcity*, *and an increase in communicable and vector-borne diseases*, *noncommunicable diseases* (*NCDs*), *and mental stress.” *[3]*.*

We will briefly discuss two issues that from the health perspective promise to become of main concern in future: air quality and the health care system. Climate change has an impact on respiratory diseases, through changes in air quality, more frequent heat-waves and an earlier onset of the pollen season in the northern hemisphere. The future impact depends on the combination of meteorological factors, and the emission of primary and secondary air pollutants. Future climate change may increase ground-level ozone pollution in Europe due to higher temperatures and weaker atmospheric circulation. The World Health Organisation reports that the changes in wind patterns and an increased desertification will increase the long-range transport of air pollutants, including aerosols, ozone, desert dust, mould spores and pesticides [4]. Higher temperatures will be more outspoken in urban settings. Ozone and particulate matter are the air pollutants with the greatest concern to health [5-7].

The health care system is not fully prepared to act in full capacity to the health problems related with climate change, in two respects. One is the uncertainty of the exact health effects related to climate change. We do know however that the impact will be substantial. It is known that the spread of diseases in Europe changes due to climate changes. Some infectious diseases may become an emerging disease in different regions of Europe, due to the fact that the meteorological climate might become milder [8-11]. A spread of disease vectors and infectious diseases in new geographical areas may be supported by rising temperatures. Changes in global trade, migration, tourism, agricultural techniques contribute to climate change and it will be difficult to state which are the most important driving forces to change [12,13]. But also non-infectious diseases show different patterns of incidence across Europe by changing climatologically based situations. An example is UV-radiation, however the links to climate change are more complex [14]. Other examples are skin cancers, cataract of the eye or allergy. Additional health issues in the population play a role with flooding, when physical trauma, infections or airway related problems arise. The number of fatalities due to catastrophic flooding events are low, even the number of acute deaths (e.g. from drowning) are small. But floods have also secondary effects: infectious diseases [15], temporary or permanent relocation of people, loss of property and related (post traumatic) stress and mental disorders [16,17], damage to infrastructure hampering and/or disrupting health services or endangering drinking water systems, and dampness and mould in houses [18] that affect housing quality for a long time. It has become clear that the health care system has to prepare itself for climate change and its related health problems.

Second, the capacity of professionals (forecasting, adapted solutions (cost, acceptance, global coverage, etc.)) which is needed to handle the future workload in the health care system is an issue. This involves complicated decisions on how to increase the capacity in the medical setting, what the priorities are regarding different medical specialities, the role of the public health sector in response to prevention or after-care with large societal disruptions after disasters or the learning needs and professionalization of the workforce.

#### Public health as a strategic notion

McMichael [19] pleads for human population health to be a sentinel criterion of environmental sustainability: *“Human population health should be the central criterion*, *and is the best long-term indicator*, *of how we are managing the natural environment.”* He identifies climate change as an important exemplar. In order to judge this strategic notion of the public health perspective on climate change, it is interesting to look at public perception research: does it strike a chord with the public? Human health aspects of climate change only recently have become a topic for public perception research [20]. Eurobarometer research on climate change e.g. did not mention health at all [21] and Eurobarometer research on health [22] does not mention climate change nor environmental issues specifically. In the Eurobarometer study on the environment as a whole though, both climate change and health are mentioned, even though not in relation to each other [23]. Even so interesting lessons can be drawn from the strategic perspective of awareness raising. In the Eurobarometer research on health [22] for example, some health issues that are mentioned by especially the youngest age groups amongst the respondents as being the most common reason for them to be receiving long term treatment are asthma (37%) and allergies (27%). Both are known to be sensitive to climate change developments. Nevertheless, the vast majority of EU respondents consider themselves to be in good health: less than one in ten (7%) say that their health is bad or very bad. This would perhaps be of concern when addressing climate change from a health perspective. Does this mean that there is no concern about climate change? Not necessarily. Climate change is perceived as the second most serious problem after *“poverty*, *the lack of food and drinking water”* and three-quarters of citizens also confirm that they take the problem very seriously [21]. From the perspective of environmental issues in general [23], climate change is of growing concern for the public: in 2007 12% more respondents cite climate change as a main environmental concern compared with 2004 whereas the shares for three other main concerns decrease. In 2007 climate change is mentioned as the second main concern: *“around a fifth of respondents associate the environment with pollution in towns and cities* (*22%*) *or to climate change* (*19%*)*.”* In 21 EU countries climate change in 2007 is even mentioned most frequently as the top environmental concern. Most respondents moreover consider the environment to play an important role in their lives, almost as important as economic and social factors [23]: *“One of the key ideas of the concept of sustainable development is that environmental and social factors should be given equal consideration with economic factors when making decisions. The concept also involves seeing these three elements as inseparable and interdependent components of human progress.* (*…*) *Respondents were asked to what extent each of these three elements affect their quality of life. A great majority of Europeans feel that all three have a great impact in their lives with more than three-quarters indicating that all three influence their daily lives either very much or quite a lot. Economic factors* (*84%*) *are seen to have the greatest impact*, *closely followed by the state of the environment* (*80%*)*.”*

Does the public in the EU feel it is well informed about climate change? According to the Eurobarometer on climate change [21]*“The extent to which respondents feel informed about certain topics related to climate change*, *i.e. their subjective level of information*, *appears to be of crucial influence on their perception of* “global warming / climate change”*.”* A little over half of the respondents in the EU seem to feel very well or fairly well informed about climate change, in respect of the causes, the consequences as well as the ways of fighting it. More than 40% though do not feel very well informed or at all informed on these aspects of climate change. The Eurobarometer on climate change [21] also asked respondents about reasons for (personally) taking action against climate change. Amongst the options respondents could choose from, health effects were not mentioned by the researchers.

Akerlof et al. [20] looked into public perceptions of climate change related health risk that resulted from recent perception research in the United States, Canada and Malta: *“When asked directly about the potential impacts of climate change on health and well-being*, *a majority of people in all three nations said that it poses significant risks; moreover*, *about one third of Americans*, *one half of Canadians*, *and two-thirds of Maltese said that people are already being harmed. About a third or more of people in the United States and Canada saw themselves* (*United States*, *32%; Canada*, *67%*), *their family* (*United States*, *35%; Canada*, *46%*), *and people in their community* (*United States*, *39%; Canada*, *76%*) *as being vulnerable to at least moderately harm by climate change. About one third of Maltese* (*31%*) *said they were most concerned about the risk to themselves and their families.”* Amongst the potential climate change related health effects mentioned by respondents in Canada and Malta, respiratory diseases, heat-related problems, cancer and infectious diseases score highly. In addition sun burn, injuries from extreme weather events and allergies are mentioned. This mirrors rather well the health effects considered by experts in relation to climate change as outlined above. Interestingly the investigators report substantial differences between the three countries with respect to more specific characteristics and consequences of climate change, such as the main consequences of climate change. Apart from differing local climate conditions, investigators also attribute this to cultural differences and difference in the personal experience of respondents. This touches upon social aspects and indeed the complexity of social aspects of climate change. We will discuss this further in the next section.

The investigators [20] recommend *“mounting public health communication initiatives that increase the salience of the human health consequences associated with climate change.”* Furthermore they point out that experience in Canada with health promotion programs attempting to motivate individuals to reduce their personal risks from climate-related hazards such as West Nile virus, smog, extreme heat and food safety, do not easily result in the adoption of health-protective behaviours. It is pointed out that in these cases climate change was not specifically mentioned in relation to these health issues, which leaves the question unanswered whether awareness raising on health problems specifically related to climate change will have more impact.

### 2) Social complexity of climate change

**Figure 1:**
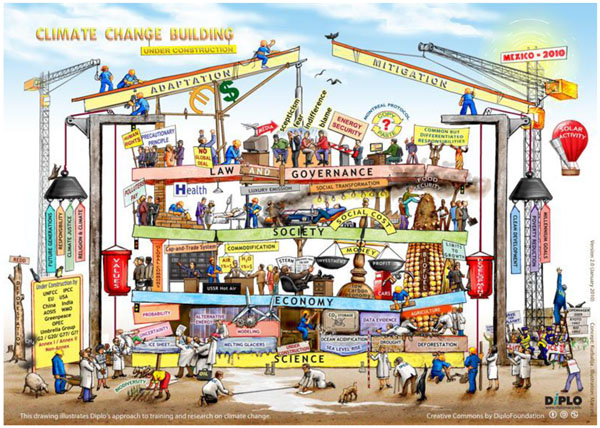
**Diplo Foundation: Climate change building **http://www.diplomacy.edu/capacity/climate

As illustrated in the previous section, climate change offers the inspiring combination of scientific complexity and societal importance. Complexity to a large extent is caused by limited scientific understanding of much climate change related issues, such as health issues. The evidence-based approach is one answer to this challenge but is widely debated because of its limited applicability to context specific real life circumstances and because complexity by definition carries the burden of imperfection. The intriguing question ‘how to interpret limited knowledge?’ remains a matter of dispute: the proof of science is in the discussion. The discussion moreover is not limited to science, to knowledge, only. The social meaning of knowledge, ‘what can or should we do with the knowledge?’ is subject to discussion also. Climate change science and policy are not only challenged by natural scientific complexity, but also by social scientific complexity. Social scientific complexity is to a large extent situated in social processes, in society, but also in science and policy making. It is increasingly realised that the importance of social processes in relation to climate change, and to sustainable technologies (such as clean energies, green chemistry, clean processes, etc.) has been undervalued, both in climate change science and technology and climate change policy making. A recent commentary in Nature [24] stresses the importance of giving increased attention to the social aspects of climate change. The social scientific complexity of climate change is beautifully illustrated by Figure 1.

Jasanoff [25] points out how main stream climate change science until now has been largely unable to connect to real social life because of its abstract character. Climate change science has difficulty in being of meaningful significance for the daily life experiences of people according to Jasanof: *“Abstraction* (*…*) *is the method by which modern science achieves its universality and heft. Science wrenches phenomena out of their specific contexts*, *makes parts meaningful independently of wholes*, *and recombines segments in ways that transgress boundaries fixed by law*, *custom*, *tradition or institutional practice.* (*…*) *It may do so with utmost honesty and care*, *but science’s products are at best images of real things*, *and much work has to be done to make the representations look as if they are the right ways of characterizing the world.”* According to Jasanoff, in order to relate better to daily life experiences, climate change science should challenge itself by connection to social processes of interpretation: *“Climate change confronts us with facts that matter crucially to the universal human destiny but that have not passed through complex processes of social accreditation on a global scale.* (*…*) *The resulting representations of the climate have become decoupled from most modern systems of experience and understanding.* (*…*) *the interpretive social sciences have a very particular role to play in relation to climate change. It is to restore to public view*, *and offer a framework in which to think about*, *the human and the social in a climate that renders obsolete important prior categories of solidarity and experience. It is to make us more aware*, *less comfortable*, *and hence more reflective about how we intervene*, *in word or deed*, *in the changing order of things.”*

### 3) Stakeholder involvement in sustainability problem solving processes

The last decades the conviction that transdisciplinarity (scientific - non-scientific actor cooperation) is vital in sustainability problem solving processes has gained support both in science and policy spheres. In an influential report for the US National Research Council Stern and Fineberg [26] stress the need for stakeholder involvement when addressing complex societal problems. An analytical deliberative approach is proposed to tackle problems more effectively. It entails a combination of on the one hand scientific methods of assessment, and on the other hand deliberation and the exchange of viewpoints between different relevant actors. Three main arguments favour stakeholder participation. The first is to broaden the support for the assessment process and the policy measures that may follow. The second is to implement democracy. The third is to broaden the knowledge base for the assessment.

An interesting example of an analytical deliberative approach is participatory backcasting [27]. Quist and Vergragt [27] trace the origins of backcasting back to the early seventies when Lovins [28] introduced the concept in the field of energy. Robinson [29] gave the concept its current name backcasting, also in relation to energy policymaking. In looking at the future, backcasting is opposite to the more traditional forecasting approach. Backcasting does not predict the future, but looks for a route towards a desirable future: how can the future we envision as desirable, be realized by actions starting today. What are the opportunities and what are the barriers? As such it is interesting in order to create a pragmatic problem solving focus, starting from ideals that perhaps look difficult to achieve under the conditions and reasoning of the present. Backcasting is an excellent approach for structuring complex issues especially when there’s a need for a long-term vision and a structural change, which is the case with many sustainability issues. Whereas the authors involved in the early days of backcasting perceived it mainly as a form of expert analysis, in the 1990’s a more participatory or interactive approach to backcasting was developed, mainly in the Netherlands [27]. We will get back to this in the methods section.

### 4) Urbanization and urban climate challenges

**Figure 2:**
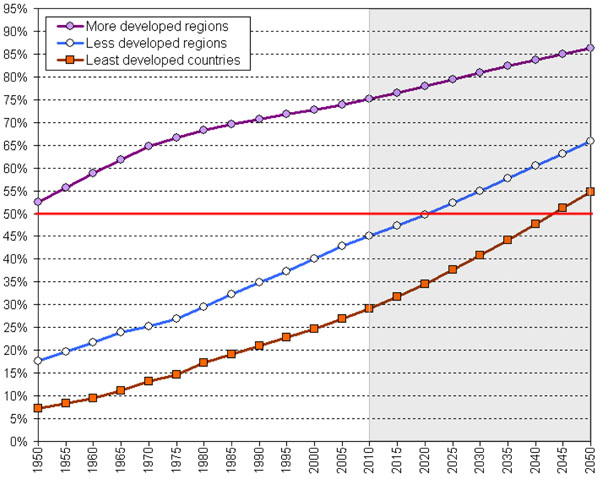
**World Urbanization Prospects: 1950 - 2050** Source: United Nations, Department of Economic and Social Affairs, Population Division: World Urbanization Prospects, the 2009 Revision. New York, 2010

**Figure 3:**
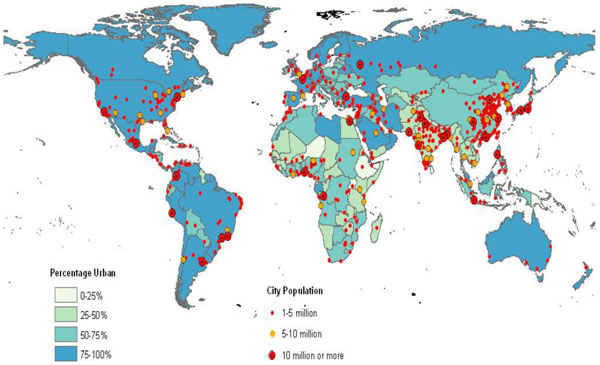
**World Urbanization Prospects: 2025** Source: United Nations, Department of Economic and Social Affairs, Population Division: World Urbanization Prospects, the 2009 Revision. New York 2010

Urban areas are of key importance for public health policies related to climate change. In major part this is because the worldwide trends of urbanization (Figure 2), in both more developed and less developed regions [30], means that by 2025 most people are expected to live in urban areas (Figure 3) [30]. The overarching challenge for these cities remains climate change, but at the same time cities must also provide good quality of life, shelter, hygiene, access to basic goods and services like drinking water, health care, or child care. A healthy environment is an important and indispensable part of quality of life in cities but works only in combination with other quality of life concerns such as social equity, income, housing, social relations, education etc. [31].

It is evident that these issues are interdependent as climate change impacts on human health (see below the example of integrated air quality and climate change) both, directly and indirectly. Directly via climate change (e.g., average and extreme temperatures, precipitation) and associated extreme weather events (floods, droughts, heat waves, fires, storms) which affect human health, in particular for vulnerable population groups. Increases in morbidity and mortality will be the consequence. Indirectly, climate change can modify and/or disrupt ecosystems and therefore have consequences for infectious diseases transmission and control.

**Figure 4:**
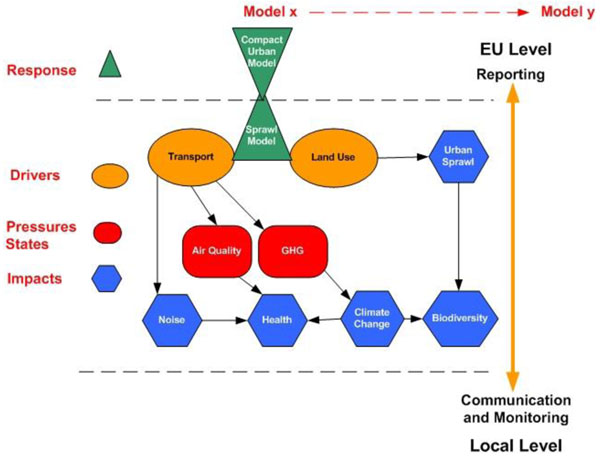
Interrelated impacts of the drivers of change

The political priority is to ensure that climate change adaptation and mitigation measures are consistent with healthy urban communities. But this is far from straightforward as managing risk and planning urban environments, involves intervening in the complex city systems, based on the interrelated impacts of the drivers of change. These relationships are illustrated in Figure 4 where drivers of urban change related to urban transport create pressures in respect of air quality and green house gas emissions that impact on human health and climate change. This complexity and interrelatedness is a prime rationale for the development of initiatives to secure the full integration of environment and health in the context of integrated urban management. Urban planning based on an integrated management approach offers a major potential to reduce the negative health impacts of climate change, preventing or minimising environmental and health problems by setting the frame for human activities and meeting human needs, such as mobility, housing, and leisure activities. The concerns of the policy-making community for integrated urban management frameworks are also reflected in the demand for new assessment methodologies for urban and regional development, which connect the political challenge of climate change mitigation and adaptation, with associated priorities to ensure healthy and economically viable urban communities. Furthermore, the pursuit of the healthy urban environment as a basis for quality of life is not only a local concern, but also a concern for all levels of governance from local to European. Planning agencies in a horizontal perspective at the local level, and in a vertical perspective from local to EU levels must combine to define and deliver appropriate solutions. Actors operating within these agencies representing a variety of stakeholder interests at local and national and European levels, must also be consulted and their views reflected in the defined solutions in order to ensure the robustness of the plan.

The Thematic Strategy on the Urban Environment promotes this integrated management approach, advocating the more effective implementation of EU environmental policies at the local level, including EU Directives on air quality, environmental noise, and urban waste water treatment, inviting Member States to support this process and encouraging local authorities to adopt integrated urban management [32]. More recently the European Commission (EC) White Paper “Adapting to climate change: Towards a European Framework for Action” [33] promotes a European framework for action in certain crucial areas including climate change and human health, and considers the necessary adaptation response of the EU and the member states in defining this framework. The framework’s main goals, include: 1) the development of the knowledge base and its expansion, 2) integration of climate adaptation in EU policy areas and increased resilience of health policies, 3) adaptation of financial mechanisms and 4) increased collaboration between EU member states.

#### The example of integrated air quality and climate change

New research identifies the potential benefits in integrating air quality and climate change policy. The study analysed the way policies addressing air pollution and climate change interact, with a focus on the way measures in one policy area affect emissions and environmental impacts in another policy area. The research predicts that accounting for the climate impact of certain air pollutants in the EU, USA and China could complement policies designed to reduce the air quality impacts of these pollutants. In Europe these relationships can be clearly identified, and according to the Green Paper on Urban Mobility [34], urban transport accounts for 70% of pollutants and 40% of greenhouse gas emissions. The outlook for urban development 2020 and beyond indicates that continued urban sprawl will trigger more transport growth, which together with the higher demand for heating and cooling of housing, will contribute to climate change, as well as regional and local air pollution and noise. All the above emphasises the potential co-benefits in integrating climate change policy and air quality legislation for certain pollutants, generating large net benefits from both perspectives. This 'integrated policy' may take several forms, for example, formally recognising pollutants as climate change agents and involving them in emissions trading schemes.

## Methods

### Backcasting

As a methodological structure of the workshop we used a backcasting format. According to Dreborg [35] backcasting is useful for issues that have the following characteristics:

• *The problem to be studied is complex*, *affecting many sectors and levels of society;*

• *There is a need for major change*, *i.e. when marginal changes within the prevailing order will not be sufficient;*

• *Dominant trends are part of the problem-these trends are often the cornerstones of forecasts;*

• *The problem to a great extent is a matter of externalities*, *which the market cannot treat satisfactorily;*

• *The time horizon is long enough to allow considerable scope for deliberate choice.*

Many sustainability issues, climate change in particular, fit this profile. A main advantage of the use of backcasting is that with this approach one is able to combine idealistic goals with pragmatics. On the one hand participants are challenged to think freely about what would be important for the future. This is possible as the method looks at a future that is rather distant, e.g. in 20 or 50 years time, thus opening horizons that might be closed by current day’s practical and ideological limitations and routines. Dreborg [35]: *“In the long term*, *the potential for man to influence development in a desired direction is relatively large. However*, *our perceptions of what is possible or reasonable may be a major obstacle to real change. The scenarios of a backcasting project may broaden the scope of solutions being considered by describing new options.”* On the other hand an advantage of the backcasting format is that from an idealistic vision pragmatic steps are considered in order to (try to) make the dreams come true. Reasoning back from the ideal future an inventory is being made obstacles and opportunities along the way and of practical steps. Another advantage of the backcasting philosophy is that it does not confine its use to a strict format, that might be more limiting than enabling. Dreborg [35] points out that there is no unequivocal method to realise a backcasting exercise: *“How you do it is not important*” and *“*(*...*) *there is no point in stipulating rules or prescribing specific methods for a creative process”.* The aim of the approach thus is to be considered more important than strict methodological rigour, thus preventing a rigor mortis of visionary creativity and good will.

### Workshop format

The workshop on integrated urban management - climate change and health impacts was organized by the FP6 EU HENVINET project. The preparation was the combined effort of an interdisciplinary team, containing expertise on climate change, environment and health, urban management and social science. It was held at the offices of Eurocities in Brussels. The HENVINET workshop had the intention to address goals identified by the European Commission (EC) White Paper *“Adapting to climate change: Towards a European framework for action” *[34] regarding the necessary adaptation responses of the EU and the member states in defining a framework for action in response to climate change, including human health:

• *Integration of climate change adaptation and health within policy frameworks at both local and EU levels;*

• *Development of the knowledge base;*

• *Fostering collaboration between relevant cities at the local level.*

Intended workshop outcomes were:

• *Identification of the opportunities and barriers to the successful integration of urban management objectives in respect of climate change adaptation and health;*

• *Identification of policy frameworks for integration of climate change adaptation and health at the local level;*

• *Development of collaboration between cities at the local level*, *and the establishment of a network to provide continuing support.*

**Figure 5:**
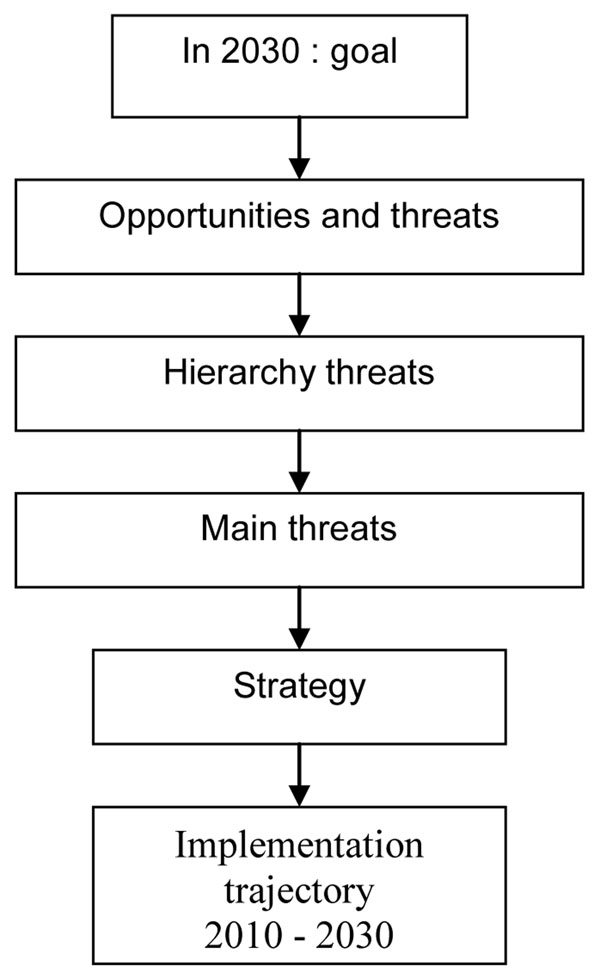
Backcasting format

As potential participants we envisaged representatives from EU cities that are engaged in climate change policy making, from EU urban management organizations such as Eurocities and experts both from science and policy making regarding the main topics of the workshop. In Figure 5 the backcasting scheme used as a format for the workshop is presented; it was adapted from the scheme used in a Belgium backcasting workshop on sustainable energy [36]. The following future goals were developed by the interdisciplinary team as food for thought for the workshop discussion:

• *Urban planning systems: In 2030 urban planning systems will have fully incorporated the necessary principles underpinning policy design and implementation to ensure that urban and regional management delivers a form of urbanisation that is fully responsive to the needs of climate change mitigation and adaptation in order to limit greenhouse gas emissions and respond to the adverse impacts of climate change. At the same time urban planning systems will have developed the capacity to ensure that decisions regarding territorial development are able to properly address and balance the complex interactions that exist between territorial development and its socio- economic impacts including health impacts.*

• *Health care systems: In 2030 the Public Health Services system in cities is fully prepared to deal with newly emerging infectious diseases and increased numbers of existing diseases caused by climate change*, *and will be doing so by realising an adaptive capacity system*, *improved early warning and diagnostics methods. This will result in prevention of public health problems and deaths related to climate change.*

• *Air quality: In 2030 cities will be able to guarantee healthy air quality in cities. In order to achieve this*, *a temperature rise in cities exceeding that of the rural areas will be prevented.*

• *Water management: In 2030 cities will be able to guarantee healthy drinking water supply in cities. In order to achieve this*, *the drinking water supply system will be safeguarded against negative impacts due to climate change.*

• *General public health in cities *(*1*)*: In 2030 cities will be able to guarantee public health at a standard independent of climate change related health problems.*

• *General public health in cities *(*2*)*: In 2030 the public health status of the population in cities will be the main indicator for assessing the success or failure of city policies in relation to climate change. By guaranteeing public health not to be victim to climate change*, *public health will be the main guiding principle for all major policy actions at city level.*

## Results

We present the workshop results in four separate parts, following the chronological order of the workshop: city perspectives, definition of a common target for 2030, discussion about opportunities, threats and other relevant issues for achieving the common goal, and an evaluation of the workshop by the participants. For an overview of the workshop participants we refer to the acknowledgement of this paper.

### City perspectives

**Figure 6:**
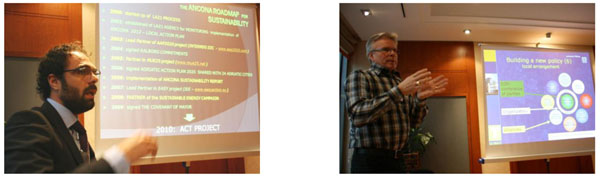
City representatives present local climate change policies to the workshop participants

**Figure 7:**
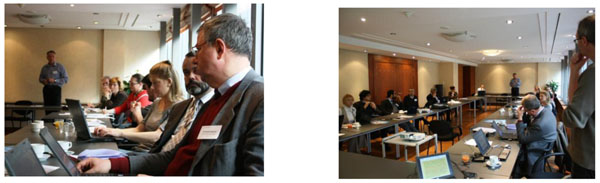
The workshop discussion.

Presentations regarding local urban climate change issues and polices were given on behalf of the cities of Bristol, Prague, Bologna, Ancona, Tilburg, and Frankfurt (Figures 6 and 7). It is evident that the cities are using a wide range of integrated management strategies, as well as focusing on different environmental topics which are based on the varying geographical and historical conditions of each city. It was also shown that many of the representative cities are linking these urban climate change issues to the local residents’ health and well-being. Outcomes of these presentations will be synthesized at the end of this section.

Bristol is encountering increasing health issues throughout the city (such as obesity, mental problems, etc.). Amongst the prime causes they have identified issues such as air pollution, urban congestion, poorly built environments (including low-density sprawl), and rising greenhouse gases. Bristol’s plan to mitigate these causes includes the need to reduce health & wealth inequality, expertise of a dedicated land-use planning team, and the goal of only producing ‘green’ buildings within the city. Bristol recognizes this as a great challenge to the city in which many of their environmental health problems are a result of cumulative impacts of smaller issues that have persisted over time.

Prague is giving special attention to poor air quality in the city centre. The city has been active in modelling the air quality problems and producing high-quality maps to better inform the public of the problems and the related health threats. The city’s main challenge has been to reduce the heat and emissions from transport, and to evaluate better transport practices which can be implemented. Prague has also made substantial efforts to analyze sustainable ways of saving energy, sustainable traffic systems, and in addition, the implementation of low emission zones for certain areas of the city centre. Prague stresses the importance of informing the public regarding the potential local health threats, and maintaining healthy dialog with residents on these threats and mitigation options.

One of the main challenges faced by the city of Bologna was how to properly evaluate energy trends since the mapping measures they employed were not giving consistent results. To overcome this issue, Bologna now uses a tool called Ecobudget which measures how much resources are available and how much the city wishes to spend. The benefit of this decision support tool is that it lets the city stay aware of what the current state is of a particular environment is, and it tracks if particular targets are reached. One of the challenges resulting from the outcome of this tool is that the city realizes it needs to have a social balance to obtain the desired goals. The mitigation measure being realized in Bologna includes the expansion of green spaces, growth of the city beneath the street level, and implementation of urban energy areas.

Ancona faces the challenges of improving mitigation measures to combat coastal erosion, and strengthening the local government and their abilities to address environmental related issues. The city recognizes that adaptation and mitigation are needed to tackle future issues associated with climate change, particularly water level rise problems. Ancona created a roadmap to sustainability in 2000, which the ACT Project (Adapting to Climate Change in Time) is currently working with. The city is also using a tool called Act project ID, which encourages an inclusive and participatory process by all local actors for development of a local adaptation plan that can forecast and mitigate environmental, social and economic impacts. This tool is a part of the cities objectives to enhance competence of local authorities, development of a baseline scenario, and general capacity building.

Tilburg has recently developed a climate program which states that in 2045 they want to have a climate neutral and climate change resistant city. In addition, Tilburg wishes to have greater control of its local arrangement so all partners (city officials, residents, and businesses) are involved in climate decisions on an equal basis so all feel the necessary responsibility and take interest in climate change policies. To accomplish these goals, the city is using the COP approach (Communities of Practice). Some of the challenges associated with Tilburg’s program is that the city needs to reduce emissions faster and use more sustainable energy sources in order to meet the 2045 goal. The COP approach relies on the use of strategic alliances which include sustainable companies, sustainable energy services, and other innovative business and organization which are willing to work together to find and implement the needed solutions. The city believes in the philosophy of “trying not to think in ‘problems’, but think in chances/solutions”.

Frankfurt is taking the approach that air pollution and climate change are different issues, even if they are inherently linked, so the city is studying these two issues separately. The city also faces the challenge of wanting to assist the micro-climate conditions but understands the need to consider the macro-climate as well. The city is currently mapping varying heat levels throughout the city. This mapping is being conducted using a scale that measures areas that contain fresh air versus areas where no vegetation can be grown. This tool results in different actions depending on location throughout the city. The city is making innovated efforts of developing a benchmark situation for urban climate (heat islands), where they started with making evaluations, which led to the generation of concrete criteria and guidelines – thus making a link between the science and the policy recommendations.

The city perspectives can be synthesized by stating that most of the cities embraced adaptation strategies in coordination with ongoing mitigation measures, and that there are strong efforts underway at the local level to embrace new alliances with a diversity of stakeholders. Cities are being faced with challenges of incorporating public health into the climate change issue. A common message resulting from the city presentations was the caution of borrowing strategies from cities with different structures (i.e. most strategies are custom tailored to the specific region they were developed for, and it may be inappropriate to simply export these strategies to new areas of different qualities).

### Definition of a common target for 2030

**Figure 8:**
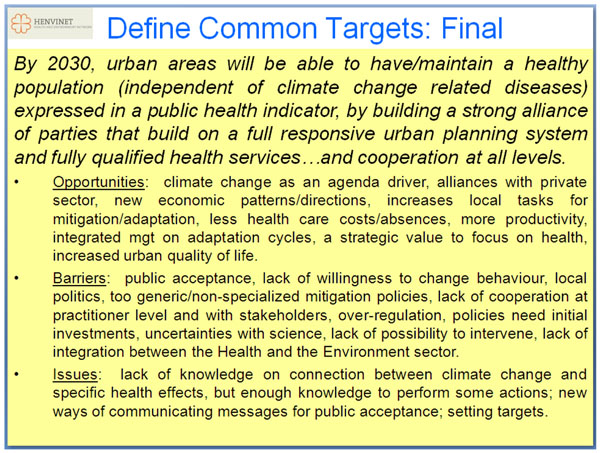
Screenshot of the work in progress

In order to trigger a discussion first the potential future goals that were developed by the HENVINET team (see in the methods section) were presented. This not only resulted in an interesting discussion (Figure 8), but also in the conception of a common target statement for the year 2030:

*By 2030*, *urban areas will be able to have/maintain a healthy population* (*independent of climate change related diseases*) *expressed in a public health indicator*, *by building a strong alliance of parties that build on a full responsive urban planning system and fully qualified health services and cooperation at all levels.*

Clearly elements from several of the potential goals presented by the workshop organizers were combined. Several specific elements were considered important. Public health is considered a key goal for urban areas; an exact qualification of a healthy population was not worked out in detail, because this was too complex for this discussion. It was recognized that careful well balanced processes should be established in which to qualify the concepts of public health and a healthy population. A clear relation was made with climate change: public health in urban areas should not be hampered by climate change, thus climate change related diseases should be prevented. This not only is complicated from a practical point of view: how to prevent climate change to have a negative impact on public health. But also it is complicated from a conceptual point of view: as was already sketched in the background section, it is very difficult to clearly distinguish climate change induced health effects from other factors that have an influence on health. Notwithstanding these complications, still the workshop participants thought it important to express public health in an indicator that would make it possible, in principle, to measure concretely to what extent policy measures would be successful in reaching this goal. For example such indicator might focus on the loss of life (more deaths, shorter life span) and loss of quality of life (due to illness).The indicator was not only discussed as an important tool for reaching the goal but also as to make public health an important indicator for climate change policies and perhaps even policy making in general, which would render it in principle the same importance as other indicators commonly used to evaluate policies, such as e.g. economic indicators. This would facilitate the weighing of public health against other policy priorities, in relation to climate change, and even with respect to policy making in general.

Another important aspect stressed by the workshop participants clearly seems to be that a procedure of working towards this target is to be realized in which integration and broad support are key factors. Cooperation by a diversity of relevant actors is considered important, as well as the integration of all relevant policy fields. This sounds idealistic and perhaps praiseworthy indeed, but, as was recognized during the workshop discussion, not at all straightforward in practice. An interesting example being that one city representative considered cooperation with health experts on the city policy level beneficial to convincing the broader public to support climate change policies from the public health perspective. A public health expert present from the same city though was clearly less optimistic about this: experience in awareness raising shows that it is not easy even from a public health perspective to convince people. One other participant gave the example of smoking: one of the worst modern health threats, yet still it is not easy to convince people to stop smoking. This also shows that from the outside (looking at public health policies) things may seem logical and straightforward whilst insiders have a much clearer view on complexities. In the next section we will discuss other issues with respect to reaching the common target of public health in relation to climate change in urban areas.

### Opportunities, obstacles and other issues

After defining a common target for 2030, the workshop discussion focussed on opportunities, obstacles and other relevant issues in the light of reaching this target (Fig 7). A diversity of issues were mentioned in relation to reaching the target presented above. A variety of opportunities in relation to economic activities and benefits were mentioned. Building alliances with the private sector is considered important in co-creating new economic patterns and directions that will be beneficial to climate change related public health issues. One example being local energy production that could both be beneficial to climate change mitigation policies and local economic development, and indirectly may result in lower health care costs because of the climate change mitigation. In general increasing local tasks for mitigation/adaptation in relation to climate change is considered important. Climate change may be used as an agenda driver for improving quality of life of which the health perspective may create new opportunities for organizing public support. The health issue may both function as a stimulator for climate change policies and as an integrator for a diversity of relevant actors and policy fields to start working together.

Though being optimistic about opportunities for reaching the target, also quite some diversity of obstacles or issues that need to be addressed were brought forward. An important issue is related to science. Science about climate change related health issues is considered to be rather limited, even though enough is known according to the workshop participants to take action. Still, specific knowledge that can lead to specific actions on a local level seems lacking. It would be beneficial to local policy making when science would be more oriented to local practices. This connects well to the conclusion drawn earlier in the background section that mainstream climate change science is too abstract with respect to everyday social life. Nevertheless redirecting science to local practice or everyday social life will not easily result in behavioral changes of local actors it is stated.

### Evaluation by the participants

The workshop was well appreciated by all participants. Several participants especially appreciated the opportunity to learn from other perspectives, both in type of background and geographically. As one participant put it: *“the event has broadened my way of thinking”.* The same participant moreover concluded that different interpretations of important issues in such workshop have a chance to be discussed: *“technical terms are not the same for all”.* Or as someone else put it: *“it takes the time to understand who is talking about what”*. Other participants also appreciated this inter- and transdisciplinary exchange of views and trying to come to a joint understanding of issues, which also was stipulated as an important part of the task ahead of reaching common goals like the one formulated in the workshop. The fact that the workshop in a one day event was not able to deliver on all aspects, especially the more concrete steps to be taken in practice on the one hand exemplified the complexity of the endeavour. Moreover, as one participant put it: *“for us it is a first approach to the problem”*. Thus, on the other hand this may also be seen as a stimulus for prolonging such activities in future, possibly in a more structured setting like a permanent expert group. All participants welcomed this idea and showed willingness to follow up on this. The structured approach of the workshop was also appreciated: *“Lots of knowledge gathered during the meeting and also because of the structured approach”*. Moreover the workshop succeeded in *“provoking critical thought on the issues”* and was characterized further as *“the meeting was like a key that opens the door”*. The workshop was seen by one participant as an *“excellent example of truly engaging stakeholders”*. Still some suggestions were made in the group about the future involvement of other actors like medical and environmental groups.

## Conclusions

The aim of the backcasting workshop discussed in this paper was bringing together the related topics regarding climate change, cities and public health that still are in need for integration. Hopefully together they will lead to strategic benefits that may outsmart the current limitations of reaching the common goal of more successful climate change policies. The workshop had the ambition to bring together a diversity of actor perspectives for exchange of knowledge and experiences, and joint understanding as a basis for future cooperation. Next to the complementarities in experience and knowledge, the mutual critical reflection was a bonus, as ideas had the opportunity to be scrutinized by others, leading to more robustness and common ground. The structured backcasting approach was helpful in integrating all of this with one common focus, embracing diversity and complexity, and stimulating reflection and new ideas. The task of both formulating a common future goal and defining a roadmap for reaching the goal was too complex for a one day workshop. Still, as one participant put it, it created hope and inspiration for future cooperation on what all participants consider to be of the utmost importance: creating a healthy future for cities in the face of climate change.

## Competing interests

The authors declare that they have no competing interests.

## Authors' contributions

HK and DL organized the workshop. PVdH and HK led the workshop. SR recorded and summarized the workshop. AB is the project manager and contributed to the workshop discussion and analysis. All authors revised the manuscript and approved the final version.

## References

[B1] Vineis P Climate change and the diversity of its health effects Int J Public Health 2010 55 81 82 10.1007/s00038-009-0092-0 19921096

[B2] Xun WW Khan AE Michael E Vineis P Climate change epidemiology: methodological challenges Int J Public Health 2010 55 85 96 10.1007/s00038-009-0091-1 19941059

[B3] Kjellstrom T Butler AJ Lucas RM Bonita R Public health impact of global heating due to climate change: potential effects on chronic non-communicable diseases Int J Public Health 2010 55 97 103 10.1007/s00038-009-0090-2 19902143

[B4] World Health Organization The solid facts on climate change and health Fact sheet 2010 Copenhagen and Parma

[B5] Engardt M Bergström R Andersson C Climate and emission changes contributing to changes in near-surface ozone in Europe over the coming decades: results from model studies Ambio 2009 38 8 452 458 10.1579/0044-7447-38.8.452 20175446

[B6] Doherty RM Heal MR Wilkinson P Pattenden S Vieno M Armstrong B Atkinson R Chalabi Z Kovats S Milojevic A Stevenson DS Current and future climate- and air pollution-mediated impacts on human health Environ Health 2009 8 Suppl 1 S8 10.1186/1476-069X-8-S1-S8 20102593 PMC2796504

[B7] Ebi K McGregor G Climate change, tropospheric ozone and particulate matter, and health impacts Cien Saude Colet 2009 14 6 2281 2293 10.1590/S1413-81232009000600037 20069198

[B8] Ready PD Leishmaniasis emergence in Europe Euro Surveill 2010 15 10 19505 20403308

[B9] Morillas-Márquez F Martín-Sánchez J Díaz-Sáez V Barón-López S Morales-Yuste M Alves de Lima Franco F Sanchís-Marín MC Climate change and infectious diseases in Europe: leishmaniasis and its vectors in Spain Lancet Infect Dis 2010 10 4 216 217 10.1016/S1473-3099(10)70052-9 20334844

[B10] Gálvez R Descalzo MA Miró G Jiménez MI Martín O Dos Santos-Brandao F Guerrero I Cubero E Molina R Seasonal trends and spatial relations between environmental/meteorological factors and leishmaniosis sand fly vector abundances in Central Spain Acta Trop 2010 115 1-2 95 102 10.1016/j.actatropica.2010.02.009 20171154

[B11] González C Wang O Strutz SE González-Salazar C Sánchez-Cordero V Sarkar S Climate change and risk of leishmaniasis in north america: predictions from ecological niche models of vector and reservoir species PLoS Negl Trop Dis 2010 4 1 e585 10.1371/journal.pntd.0000585 20098495 PMC2799657

[B12] Morris GP Ecological public health and climate change policy Perspect Public Health 2010 130 1 34 40 10.1177/1757913909354149 20333948

[B13] Moore MN Kempton PD A synopsis of the Joint Environment and Human Health Programme in the UK Environ Health. Suppl 2009 1 S1 10.1186/1476-069X-8-S1-S1 PMC2796487 20102576

[B14] Environmental Effects Assessment Panel Environmental effects of ozone depletion and its interactions with climate change: progress report, 2009 Photochem Photobiol Sci 2010 9 3 275 294 20301813 10.1039/b923342n

[B15] Ivers LC Ryan ET Infectious diseases of severe weather-related and flood-related natural disasters Curr Opin Infect Dis 2006 19 5 408 414 10.1097/01.qco.0000244044.85393.9e 16940862

[B16] Carroll B Morbey H Balogh R Araoz G Flooded homes, broken bonds, the meaning of home, psychological processes and their impact on psychological health in a disaster Health Place 2009 15 2 540 547 10.1016/j.healthplace.2008.08.009 18996730

[B17] Lalande G Maltais D Robichaud S Disaster victims of the 1996 Saguenay floods: problems experienced and psychological consequences Sante Ment Que 2000 25 1 95 115 [Article in French] 18253573

[B18] Barbeau DN Grimsley LF White LAE El-Dahr JM Lichtveld M Mold exposure and health effects following hurricanes Katrina and Rita Annu Rev Public Health 2010 31 165 178 10.1146/annurev.publhealth.012809.103643 20070193

[B19] McMichael AJ Human population health: sentinel criterion of environmental sustainability Current Opinion in Environmental Sustainability 2009 1 101 106 10.1016/j.cosust.2009.07.001

[B20] Akerlof K DeBono R Berry P Leiserowitz A Roser-Renouf C Clarke KL Rogaeva A Nisbet MC Weathers MR Maibach EW Public Perceptions of Climate Change as a Human Health Risk: Surveys of the United States, Canada and Malta Int. J. Environ. Res. Public Health 2010 7 2559 2606 10.3390/ijerph7062559 20644690 PMC2905567

[B21] European Commission Europeans’ attitudes towards climate change. Eurobarometer 2008 http://ec.europa.eu/public_opinion/archives/ebs/ebs_300_full_en.pdf

[B22] European Commission Health in the European Union. Eurobarometer 2007 http://ec.europa.eu/health/ph_publication/eb_health_en.pdf

[B23] European Commission Attitudes of European citizens towards the environment. Eurobarometer 2008 http://ec.europa.eu/public_opinion/archives/ebs/ebs_295_en.pdf

[B24] Smith K The wisdom of crowds Nature reports climate change 2009 3 89 91

[B25] Jasanoff S A New Climate for Society Theory, Culture & Society 2010 27 2–3 233 253 http://tcs.sagepub.com/content/27/2-3/233

[B26] Stern PC, Fineberg HV Understanding risk: information decisions in a democratic society 1996 National Research Council, National Academy Press, Washington D. C.

[B27] Quist J Vergragt P Past and future of backcasting: The shift to stakeholder participation and a proposal for a methodological framework Futures 2006 38 1027 1045 10.1016/j.futures.2006.02.010

[B28] Lovins A Energy strategy: the road not taken? Foreign Affairs 1976 55 63 96

[B29] Robinson J Energy backcasting: a proposed method of policy analysis Energy Policy 1982 10 337 344 10.1016/0301-4215(82)90048-9

[B30] United Nations Department of Economic and Social Affairs Population Division World Urbanization Prospects, the 2009 Revision 2010 New York

[B31] European Environment Agency Ensuring quality of life in Europe's cities and towns. Tackling the environmental challenges driven by European and global change EEA Report No 5/2009 Copenhagen

[B32] European Commission Thematic Strategy on the urban environment COM(2005) 718 final 2006 Brussels

[B33] European Commission White Paper: Adapting to climate change: Towards a European framework for action COM(2009) 147 final 2009 Brussels

[B34] European Commission Green Paper: Towards a new culture for urban mobility COM (2007) 55 2007 Brussels

[B35] Dreborg KH Essence of backcasting Futures 1996 28 813 828 10.1016/S0016-3287(96)00044-4

[B36] Keune H Bongaerts V Verbruggen A Goorden L A future for nuclear power in Belgium? ENER bulletin 2003 European Network for Energy Economics Research

